# 

**Published:** 1917-01-20

**Authors:** 


### CONTENTS.

PAGE

**War, Alcohol, and Criminals** ... ... ... ... 311

**Churches and Charity Funds** ... ... ... ... 312

**Hospital and Institutional News** ... ... ... 313

The Ownership of Extracted Bullets—A Case Worthy of Portia—Grimsby Hospital Records—The Closing of an Out-Patient Department at Lincoln—Large Gifts to New War Hospital—Food Prices and Patients' Fees—Newman on the Hospital Ideal—February 14, 1917—Expanding Collections for Voluntary Hospitals—Caution in Cornwall—The Barnstaple Smallpox Hospital Ship—The Seamen's Hospital—The Railway Fares of Hospital Workers—The Call from the Colours—The Royal Earlswood Institution—A Patients' Book—Gardening Under Medical Supervision—Cabbages and Potatoes on a Hospital Site—"Have You Seen Our Vegetable Beds?"—Potato Substitutes in Hospital Diet—"Recuperative Hostels"—State Drug Depôt at Sydney—The Scarcity of Teachers of Remedial Exercises—Medical Status of Christian Scientists—Ward Wit and "Fowl Play"—Hospital Music—The Importance of a Standard—"What Makes an Army."

**How Blinded Soldiers are Cared For** ... ... 319

By Mr. Arnold Lawson, F.R.C.S., Ophthalmic Surgeon, Middlesex Hospital and St. Dunstan's.

**The Problem of Population...** ... ... ... 321

By a Practitioner at the Front.

PAGE

**Medical Home-Truths** ... ... ... ... ... 322

II. Medicine in General.

**Reform in Tuberculosis Work** ... ... ... 323

II. The Sanatorium.

**Public Health Administration** ... ... ... 324

The Rise of State Preventive Medicine.

**National Insurance Act Developments...** ... 325

Pension Problems.

**Legal Notes for Institutional Workers** ... 326

**Homeland Workers in War Time** ... ... 327

The Remedy of Recognition an Imperative Duty. Round the Hospitals.

Overworked Principal Matrons—A "Make-believe" Infant—Meetings in Dublin and Belfast—Suggested Royal Charter—Irish Nurses Organising—Irish College Committee Appointed.

**The Editor's Letter-Box** ... ... ... ... 329

Churches and Charity Funds—Irish Local Government Boards and Disloyal Boards of Guardians—Pressing Need at the Front for Mufflers.

**Matrons' and Sisters' Appointments** ... ... iv

**Passing Events and Topics...** ... ... ... iv

**The Institutional Worker** ... .. (Suppl.) 1

Precautions in Theatre Work at Operations. Questions for January.

